# Early increased neutrophil-to-lymphocyte ratio is associated with poor 3-month outcomes in spontaneous intracerebral hemorrhage

**DOI:** 10.1371/journal.pone.0211833

**Published:** 2019-02-07

**Authors:** Jie Qin, Zhu Li, Guangming Gong, Hongwei Li, Ling Chen, Bo Song, Xinjing Liu, Changhe Shi, Jing Yang, Ting Yang, Yuming Xu

**Affiliations:** 1 Third Department of Neurology and Key Disciplines Laboratory of Clinical Medicine of Henan Province, The First Affiliated Hospital of Zhengzhou University, Zhengzhou, Henan, P. R. China; 2 Department of Immunology, College of Basic Medical Sciences, Zhengzhou University, Zhengzhou, Henan, P. R. China; 3 Department of Neurosurgery, The First Affiliated Hospital of Zhengzhou University, Zhengzhou, Henan, P. R. China; Massachusetts General Hospital, UNITED STATES

## Abstract

The aim of this study was to evaluate the association of dynamic neutrophil-to-lymphocyte ratio (NLR) with 3-month functional outcomes in patients with sICH. We retrospectively identified 213 consecutive patients with sICH hospitalized in The First Affiliated Hospital of Zhengzhou University from January 2017 to May 2018. Patients were divided into functional independence (FI) or unfavorable prognosis (UP) groups based on 3-month outcomes. Admission leukocyte counts within 24 hours of symptom onset were obtained, and the recorded fraction, of which the numerator is neutrophil and the denominator is lymphocyte, as NLR0. Determined NLR1, NLR3, NLR7, and NLR14 were recorded on day 1 (n = 77), day 3 (n = 126), day 7 (n = 123), and day 14 (n = 105), respectively. The relationships between dynamic NLR or leukocyte counts and clinical features were evaluated using Spearman’s or Kendall’s correlation analysis. Logistic regression analyses were used to identify the risk factors for unfavorable 3-month prognosis. The patients’ dynamic NLR was positively associated with the National Institutes of Health Stroke Scale, ICH score, and hematoma volume at admission, while inversely correlated to the onset GCS score and FI at 3-month follow-up. Furthermore, higher NLR or lower absolute lymphocyte count obtained at admission was independently risk factor for UP at 3 months (adjusted odds ratio [OR]: 1.06, 95% confidence interval [CI]: 1.003, 1.12; OR: 0.41, 95% CI: 0.18, 0.94, respectively). In conclusion, higher NLR and lower lymphocyte counts at early stages were predictive of 3-month unfavorable outcomes in sICH patients.

## Introduction

Spontaneous intracerebral hemorrhage (sICH) accounts for approximately 70% of hemorrhagic strokes, with high mortality, morbidity, and disability rates [1]. It is one of the most devastating diseases globally, especially in developing countries [2]. For improving the outcomes in sICH, existing surgical or medical treatments are insufficient [3, 4].

Various risk factors in clinical predictive models based on ICH score have been studied to identify sICH patients with unfavorable prognoses, including age, Glasgow Coma Scale (GCS) score, ICH volume, intraventricular hemorrhage (IVH), and infratentorial origin of ICH [5, 6]. However, risk factors which just typically based on clinical information, such as symptoms and images, contributing to ICH score actually omitted lab biologic elements predictive values for sICH clinicopathologic features [7]. Experimental and clinical evidence has demonstrated that systemic immune-inflammatory responses play an important role in secondary brain injury after sICH onset [8, 9]. The developing systemic inflammatory response is characterized by elevated numbers and activation of inflammatory-related cells, such as neutrophils, monocytes; decreased lymphocyte counts; and the release of various inflammatory cytokines and mediators [8–10]. After local brain injury, a cascade of pro-inflammatory cytokines and chemokines, such as TNF-α and IL-1β, are released by activated microglia, epithelium, and other cells in brain [8, 11]. These mediators attract peripheral neutrophils and lymphocytes, which gradually migrate and aggregate around the hematoma within a few hours; and then release various pro-inflammatory or anti-inflammatory cytokines, for recognized examples of nitric oxide synthase (iNOS) and matrix metalloproteinase (MMP)-9 [12]. This sterile inflammatory cascade reaction contributes to edema and exacerbates brain injury, which affects severity and outcomes of ICH [8]. Cytokine-related inflammatory and immune profiles in ICH patients are not reflected in most clinical predictive models with well-entrenched ICH scores, might due to a loss of prediction accuracy arising from the complexity of cytokine networks *in vivo*.

The peripheral neutrophil-to-lymphocyte ratio (NLR) is a fraction, of which the numerator is neutrophil and the denominator is lymphocyte. Compared with conventional cytokine-related inflammatory biomarkers, NLR is more easily accessed from blood cell counts and accurately reflects immune and inflammatory status. Its superior predictive value for clinical outcomes has been demonstrated in patients with major cardiac events, ischemic stroke, diabetes mellitus, chronic obstructive pulmonary disease, cancers, sepsis, infectious pathologies, and liver failure [13–18]; however, the association between NLR and clinical outcomes of sICH remains controversial. Previous studies premise that high NLR reflects an elevation of neutrophils and down-regulation of lymphocytes in peripheral blood, is an independent predictor of poor clinical short-term outcomes after sICH [19–22]. However, other studies have demonstrated that NLR has no significant effect on mortality, and 30- or 90-day poor outcomes [23, 24].

In this study, we examined the predictive value of peripheral dynamic NLRs with 3-month functional outcomes after acute sICH onset to investigate whether such initial immune imbalance, measured by routine bloods, is associated with intermediate-term outcomes in sICH. We evaluated the clinical data of patients following sICH and monitored changes in peripheral absolute neutrophil and lymphocyte counts.

## Methods

### Study population

This retrospective study consecutively enrolled hospitalized patients with acute sICH at The First Affiliated Hospital of Zhengzhou University from January 2017 to May 2018. Inclusion criteria were: 1) aged between 18–60 years old; 2) immediate routine blood sampling after hospital admission and verification by head computed tomography (CT) scan within 24 hours from the onset of stroke-like symptoms; 3) absence of secondary causes of ICH (including trauma, ruptured malformed cerebral vessels, hemorrhagic transformation after cerebral infarction, drug-induced ICH, abnormal coagulation, or other causes detected during hospital procedures); 4) absence of infection for 14 days and history of stroke for 6 months; 5) absence of cancer, autoimmune disease, severe hepatic or renal diseases, or use of steroid immunomodulatory treatments. A proportion of patients had routine blood reexaminations and were chosen from hospitalization day 1, 3, 7, or 14, respectively. Only 26% of sICH patients were registered in the intensive care unit (ICU). All patients were managed according to the 2015 American Heart Association/American Stroke Association (AHA/ASA) guidelines [1]. Differences in ward sources were further adjusted in the multivariate logistic regression models.

This study was conducted in accordance with the guidelines of the Helsinki Declaration and the recommendations of The Ethics Committee of The First Affiliated Hospital of Zhengzhou University. The Ethics Committee of The First Affiliated Hospital of Zhengzhou University approved the study protocol (2018-LW-031), and waived written informed consent because of the retrospective nature of the study. All subjects were anonymized, authors had no access to information that could identify individual participants during or after data collection.

### Laboratory, radiological, and clinical parameters

Total white blood cells (WBC), absolute neutrophil count (ANC), absolute lymphocyte count (ALC), and relevant computed NLR at admission and hospitalization day 1, 3, 7, or 14 were recorded and calculated from routine bloods using a COULTER LH780 Hematology Analyzer (Beckman Coulter, Inc, Orange County, CA, USA). Age, sex, time from onset to sampling, past medical history, baseline radiological collections, and clinical parameters were documented in medical records.

sICH was diagnosed based on neurological deterioration and confirmed with a head CT by two experienced neurologists. The hematoma volume at admission was measured using the ABC/2 method (where A is the greatest hemorrhage diameter by CT, B is the diameter 90° to A, and C is the approximate number of CT slices with hemorrhage multiplied by the slice thickness) [25]. The large group was defined as having a hematoma volume >30 cm^3^. There was a large amount of variability in ICH severity and neurological dysfunction; therefore, ICH score (measured by age, hematoma volume, location, GCS score, and IVH), and National Institutes of Health Stroke Scale (NIHSS) scores were used. Patients with fever after admission (due to pneumonia, urinary tract infection, intracranial infection, or other identified reasons), CRP >10 mg/L, and procalcitonin >0.046 ng/mL within 14 days of hospitalization were classified into the infectious group [26]. We obtained patients’ follow-up information regarding mortality and modified Rankin Scale (mRS) score 3 months after discharge. The functional independence (FI) group included patients with an mRS score of 0–2. The unfavorable prognosis (UP) group included patients with an mRS score of ≥3 or who had died.

### Statistical analyses

Skewed distribution variables are described by median (inter-quartile range) or categorical variables with number (%). The Mann-Whitney U or χ^2^ test (or Fisher exact test when the expected value was <5) was used to compare different subgroups. Correlations between NLR (including at admission, days 1, 3, 7, and 14) and admission NIHSS score, ICH score, hematoma volume, and infectious condition were evaluated using Spearman’s correlation coefficient (r) or Kendall’s relation analysis. We used a clinically significant threshold of *p* < 0.1 to identify candidate variables for inclusion in logistic regression models in all single factor analyses. In multivariate logistic regression analyses, the likelihood ratio test with α value of 0.05 was used to test statistical significance. We report associations as odds ratios (ORs) with corresponding 95% confidence intervals (CIs) in logistic regression models. SPSS 18 (SPSS Inc., Chicago, IL, USA) and GraphPad Prism 6 (GraphPad Software, San Diego, Calif) were used for all statistical analyses.

## Results

### Baseline characteristics of patients

We enrolled 213 patients with sICH in this study. The median age was 50 years old, and 73% were male. NLR at admission was 7.3 (4.3–15.7). The proportion of patients discharged for death was 8.2%. Mortality was 11.5% at 3 months (Table 1).

**Table 1:** Baseline characteristics of patients (n = 213).

Variable	Value
Age, median (IQR), y	50 (46–55)
Male sex, %	73.7
Time from onset to sampling, h	4 (3–7)
Previous medical history	
Coronary heart disease, %	4.2
Hypertension, %	72.8
Diabetes mellitus, %	9.4
Hyperlipidemia, %	33.8
Historical mRS score, median (IQR)	0
History of stroke, %	
Infarction	8.5
Hemorrhage	8.9
Both	0.9
Hospital characteristics	
Intensive care unit (ICU), (%)	26
Endotracheal intubation, (%)	28
Nasogastric feeding tube, (%)	55
Catheter, (%)	74
Operation, (%)	43
Infection within 14th day, (%)	52
Duration of hospitalization, d, %	
≤7	17.9
7–14	21.1
>14	62.0
Baseline CT findings	
Baseline ICH volume, median (IQR), cm^3^	17 (7.0–31.4)
Larger ICH volume (>30 cm^3^), %	28.2
Infratentorial, %	11.3
Intraventricular hemorrhage, %	18.8
Admission laboratory values	
Total white blood cells (WBC), 1000/mm^3^	10.6 (8.1–14.6)
Absolute neutrophil count (ANC), 1000/mm^3^	8.8 (5.9–12.7)
Absolute lymphocyte count (ALC), 1000/mm^3^	1.2 (0.8–1.6)
NLR0^a^	7.2 (4.2–15.7)
Absolute monocyte count (AMC), 1000/mm^3^	0.5 (0.3–0.7)
Admission functional assessment	
ICH score, median (IQR)^b^	1 (0–2)
NIHSS admission score, median (IQR)	10 (5–12.3)
Glasgow coma scale, median (IQR)	13 (7–15)
Mortality at discharge, %	8.5
3-month follow-up information	
Mortality, %	11.7
mRS scores, median (IQR)	1 (0–2)
Functional Independence, (FI), mRS (0–2), n, %	74

GCS, Glasgow Coma Scale; ICH, intracerebral hemorrhage; mRS; modified Rankin scale; NLR, neutrophil-to-lymphocyte ratio; NIHSS, National Institute of Health Stroke Scale

^a^NLR0, neutrophil-to-lymphocyte rate at admission. Reasonably relevant NLRs on days 1, 3, 7, and 14 are NLR1, NLR3, NLR7, and NLR14 respectively.

^b^ICH score, total 6 marks, include GCS score (0–2), hematoma volume (0–1) and location (0–1), IVH (0–1) and age (0–1).

### NLRs are associated with ICH admission severity

Spearman’s correlation analysis revealed that NLRs were moderately positively associated with admission NIHSS score, ICH score, and hematoma volume; but negatively correlated with GCS score (*p* < 0.05 for all analyses; Table 2). Furthermore, Kendall’s correlation analysis revealed no correlations between NLRs and volume locations (all p > 0.05, S1 Table)

**Table 2:** NLRs are associated with the severity of ICH.

	Spearman’s Correlation, r, *p* values			
	NIHSS score	ICH score	GCS score	Hematoma volume
NLR0	0.386, <0.001	0.487, <0.001	−0.373, <0.001	0.507, <0.001
NLR1	0.232, 0.049	0.325, 0.004	−0.238, 0.03	0.378, 0.001
NLR3	0.295, 0.001	0.423, <0.001	−0.383, <0.001	0.473, <0.001
NLR7	0.272, 0.002	0.452, <0.001	−0.353, <0.001	0.463, <0.001
NLR14	0.285, 0.003	0.363, <0.001	−0.347, <0.001	0.449, <0.001

ICH, intracerebral hemorrhage; NLR, neutrophil-to-lymphocyte ratio

### Relationship of NLR profile with functional outcomes

**Fig 1:**
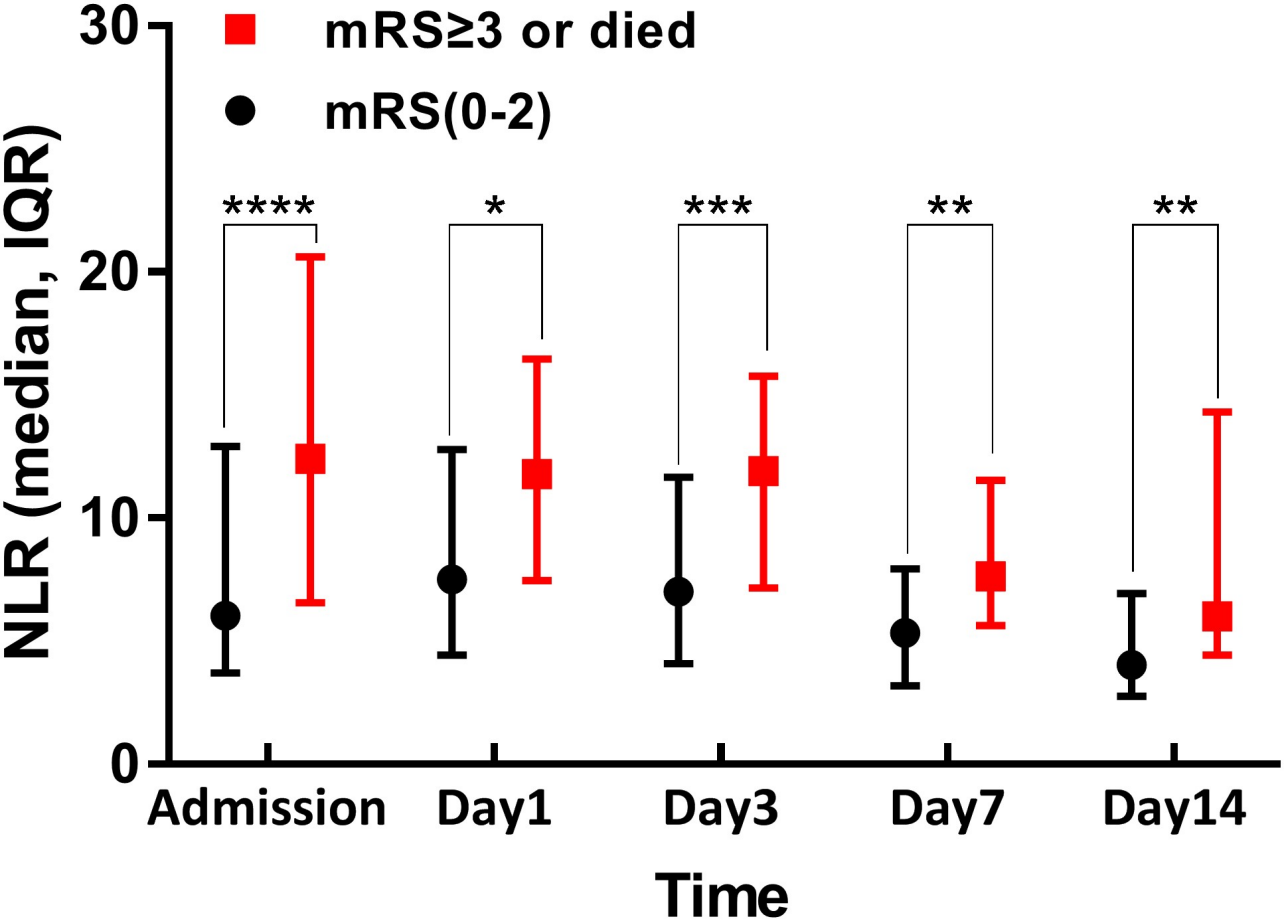
Dynamic profile of neutrophil-to-lymphocyte ratio (NLR) in spontaneous intracerebral hemorrhage (sICH) patients from admission to 14th day of hospitalization. Black bar represents functional independence group; red bar represents unfavorable prognosis group. There were significant differences between both groups at each time point (*p < 0.05; **p <0.01; ***p < 0.001; ****p < 0.0001).

A decreasing trend was observed in the medians of all NLRs (including NLR0, NLR1, NLR3, NLR7, and NLR14) from admission to the 14th day of hospitalization (Fig 1). All NLR medians at each time point in the UP group were higher than the FI group during hospitalization (Fig 1). In addition, these NLRs were negatively correlated with functional independence at 3 months (Kendall’s relationship analysis; Table 3).

**Table 3:** Patients in functional independence (FI) and mRS (0–2) groups at 3 months after intracerebral hemorrhage.

NLRs	Total (n)	Functional independence (FI), mRS (0–2) (n)	Correlation (Kendall’s tau-b, *p* values)
NLR0	213	158	−0.223, <0.001
NLR1	76	44	−0.247, 0.009
NLR3	126	83	−0.269, <0.001
NLR7	123	88	−0.237, 0.001
NLR14	105	80	−0.259, 0.001

mRS, modified Rankin Scale; NLR, neutrophil-to-lymphocyte ratio

### NLR0 and admission ALC, but not WBC or ANC, are associated with 3-month outcomes

Candidate variables from the univariate logistic regression (S2 Table), such as age, sex, hyperlipidemia, NIHSS score, hematoma volume with *p* < 0.1 or reasonable clinical significance, were chosen for the subsequent multivariate logistic regressions.

In model 1, multivariate logistic regression analysis revealed that the risk of unfavorable prognosis increased by 1.06-fold per one unit added value for NLR0. In model 2, an ALC increase of 1000/mm^3^ with adjusted candidate confounders decreased the risk of unfavorable prognosis by 0.41-fold at 3 months (Table 4).

**Table 4:** Multivariate logistic regression analyses of admission NLR0, ALC characteristics of 3-month functional independence (FI, mRS 0–2).

Model 1	OR	95% CI	*p*
Age	0.96	0.89, 1.03	0.262
Male sex	1.94	0.64, 5.87	0.243
Hyperlipidemia	0.60	0.19, 1.91	0.382
A history of stroke	0.80	0.23, 2.80	0.731
Intensive care unit (ICU)	0.34	0.09, 1.35	0.126
Endotracheal intubation	0.29	0.07, 1.13	0.074
Nasogastric feeding tube	0.31	0.06, 1.67	0.173
Operation	0.79	0.19, 3.35	0.754
Infection	0.18	0.03, 0.94	0.042^a^
Duration of hospitalization, d			
≤7			0.614
7–14	0.36	0.05, 2.75	0.326
>14	0.70	0.15, 3.20	0.645
Infratentorial	1.26	0.18, 8.98	0.818
Intraventricular hemorrhage	0.62	0.18, 2.19	0.457
Larger ICH volume (>30 cm^3^)	0.19	0.05, 0.67	0.010^a^
NIHSS admission score	0.83	0.74, 0.94	0.003^a^
Glasgow coma scale	0.91	0.75, 1.10	0.343
NLR0	1.06	1.003, 1.12	0.039^a^
Model 2	OR	95% CI	*p*
Age	0.95	0.88, 1.02	0.136
Male sex	1.59	0.49, 5.10	0.440
Hyperlipemia	0.65	0.20, 2.12	0.479
A history of stroke	0.73	0.21, 2.50	0.612
Intensive care unit (ICU)	0.40	0.10, 1.60	0.192
Endotracheal intubation	0.40	0.10, 1.56	0.186
Nasogastric feeding tube	0.29	0.05, 1.52	0.142
Operation	0.82	0.19, 3.45	0.782
Infection	0.19	0.04, 0.93	0.041^a^
Duration of hospitalization, d			
≤7	0.683		
7–14	0.42	0.06, 3.14	0.395
>14	0.69	0.16, 3.02	0.617
Infratentorial	1.36	0.17, 10.59	0.771
Intraventricular hemorrhage	0.68	0.18, 2.58	0.573
Larger ICH volume (>30 cm^3^)	0.21	0.06, 0.74	0.016^a^
NIHSS admission score	0.82	0.72, 0.93	0.002^a^
Glasgow coma scale	0.92	0.76, 1.11	0.382
ALC, 1000/mm^3^	0.41	0.18, 0.94	0.036^a^

ALC, absolute lymphocyte count; ICH, intracerebral hemorrhage; mRS, modified Rankin scale; NIHSS, National Institutes of Health Stroke Scale

^a^*p <* 0.05, variables that were independent predictive factors for 3-month outcomes of acute ICH.

Hematoma volume >30 cm^3^, increased NIHSS score, and infectious condition were also independent risk factors for outcomes at 3 months, after adjusting for other variables (Table 4, model 1 and model 2).

In contrast, after adjusting for the same confounders, WBC (OR = 1.11), ANC (OR = 1.14) at admission, did not produce statistically significant values for 3-month outcomes (S3 Table).

### Early decrease in ALC is associated with unfavorable ICH prognosis

**Fig 2:**
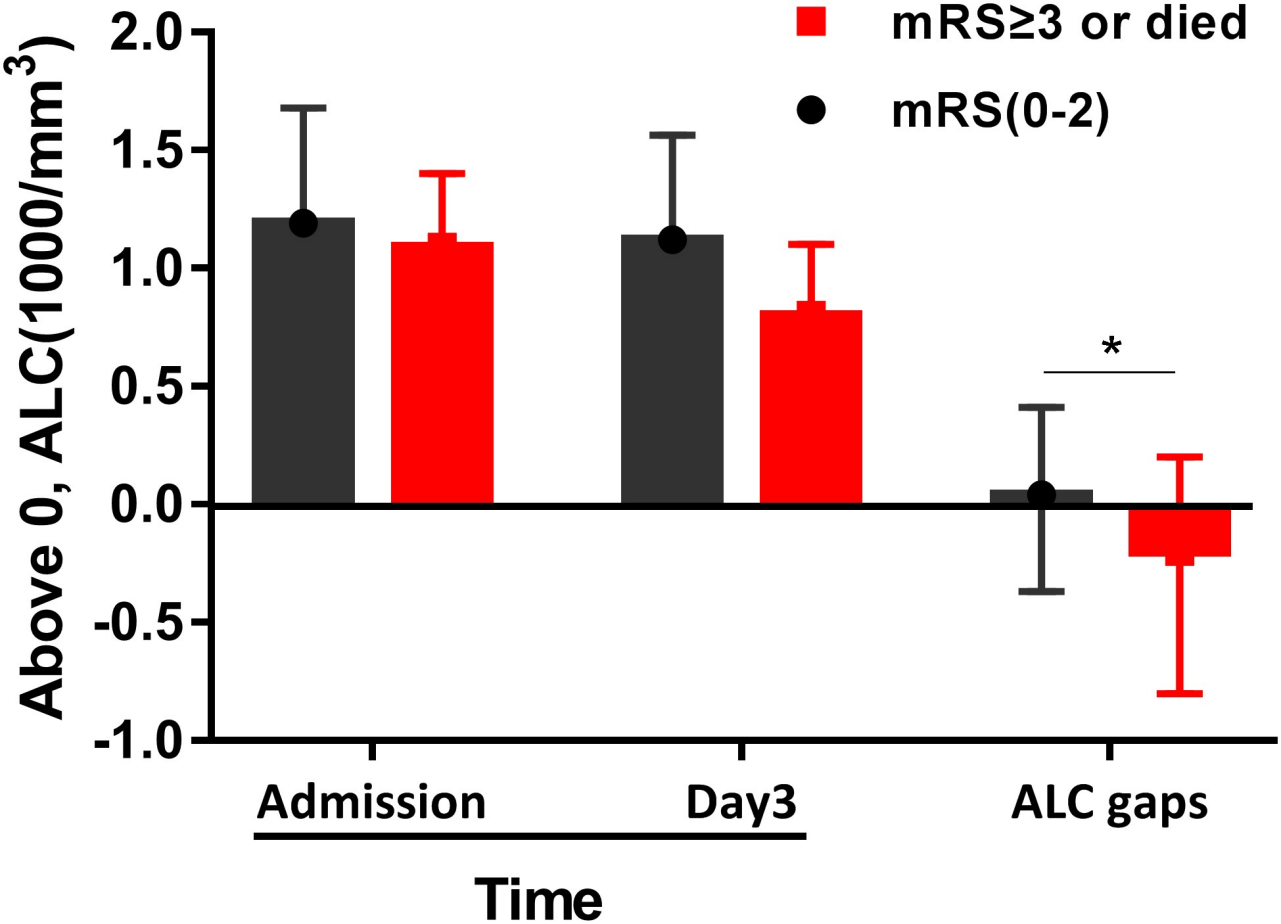
The absolute lymphocyte count (ALC) changes in spontaneous intracerebral hemorrhage (sICH) patients from admission to 3^rd^ day of hospitalization. Black bar represents functional independence group; red bar represents unfavorable prognosis group. The descending difference in ALC within 3 days of hospitalization (represented by ALC gaps) between two groups was statistically significant (*p < 0.05).

Dynamic monitoring of early leukocyte counts, in 126 patients who were randomly chosen for routine blood reexamination on day 3, revealed that 83 FI and 43 UP individuals at the 3-month time point exhibit different ALC reductions (represented by ALC gaps) from admission to day 3 (*p <* 0.05; Fig 2).

## Discussion

The main findings of our study were that higher NLR or lower ALC in patients with sICH during the early stage of hospitalization was independently associated with 3-month outcomes, after adjusting for confounders, including NIHSS score, hematoma volume on admission, and infections from admission to day 14 of hospitalization.

Acute sICH causes local brain injury characterized by aseptic necrosis. As a result of the inflammatory cascade, innate cells rapidly infiltrate the hematoma and surrounding site through a highly permeable BBB within 6–24 hours, reaching a peak at 2–3 days [27]. These innate cells clear necrotic tissue and fight potential ICH-induced infection or autoimmune reactions [28]. Neutrophils are one of the first immune cells that migrate to the hematoma and play a crucial role in acute and innate inflammatory reactions [29, 30]. In most situations, the intensity of neutrophil response represents acute and active inflammatory reactions for the initial clearance of potential pathogens before the onset of the adaptive immune response. Nevertheless, circulating neutrophils recruited to the site of cerebral injury further contribute to disruption of the BBB, neurovascular units, and other cerebral tissues *via* release of neutrophil-derived factors, including proteases, reactive oxygen species (ROS), myeloperoxidase, elastase, cathepsin G, and inflammatory mediators, such as cytokines and chemokines [27, 30, 31].

The proliferation of peripheral lymphocytes in adaptive immunity remains depressed at 24 hours, extends beyond 4–7 days after ICH [32]. In addition, the rapid decrease in peripheral lymphocyte counts at the acute stage of sICH is due to lymphocyte infiltration into the brain [33, 34]. Excessive recruitment of lymphocytes and neutrophils from peripheral blood due to ICH-induced intracerebral damage can lead to a state of temporary immunodeficiency [35]. Stroke-induced immunodepression may contribute to the decrease in peripheral lymphocytes and reduce inflammatory-mediated cerebral damage by attenuating over activation of the inflammatory cascade. Therefore, immunodepression post-stroke inversely increases susceptibility to infection and is detrimental to clinical outcomes [36, 37].

Recently, lymphocytes have been shown to play dual roles in inflammatory attack or immune defense after stroke. Lymphocytes that infiltrate into brain tissue potentiate cerebral inflammation and brain injury in animal ICH models [33]. In a clinical trial, fingolimod, a drug that reduces the cycling pool of T-lymphocytes, was administered to ICH patients with a small hematoma volume (<30 mL) no later than 72 h after onset [38]. The authors reported a reduction in brain edema and improved 3-month outcomes. Furthermore, developing lymphocytopenia (lymphocyte count <10^9^/L) affected the 3-month outcomes of sICH; patients with ICH who had lymphocytopenia within the first 5 days of admission exhibited poorer 3-month outcomes compared with those with lymphocytopenia on admission [39]. In our study, compared with patients who showed a slowly descending tendency of ALC, those with a sharply descending ALC over the first 3 days post-acute sICH exhibited larger hematoma volumes and worse 3-month outcomes. These results suggest that single and variable inflammatory indicators cannot fully reflect the host’s immune status. This limits their utility in predictive models for effective evaluation of stroke severity and prognosis.

The dynamic profiles of NLRs represent ongoing changes in two leukocyte subsets. The link between innate and adaptive immune responses in patients with acute sICH reflects their inflammatory status and intensity of systemic immune responses, especially in the early stage of hospitalization. Currently, studies that focus on chronic inflammation, which may be the cause and result of diabetes mellitus, at the gene level in inflammation-associated cells and tissues suggest that a significant increased NLR level could be used as a predictive biomarker of type 2 diabetes mellitus [18]. Equally, many studies have demonstrated the same relationship between a higher NLR and worse ischemic stroke outcomes [40–42]. However, data from the limited number of studies that have focused on ICH stroke are not sufficient to describe the true profiles of NLR at present. Consistent with Sun et al. patients with acute ICH exhibit an association between NLR at admission and stroke severity [24]. Further, our results suggested that NLRs within 2 weeks after ICH onset are associated with NIHSS score, ICH score, hematoma volume, GCS score, and infectious complications. This indicates that neutrophils and lymphocytes associated with the ICH severity had important roles in ICH-induced brain injury and repair during this time period.

Clinical outcomes post-ICH are affected by various factors, such as blood pressure and drug administration. In-hospital infection complications are a leading cause of morbidity and mortality in patients with ICH [43]. Further, higher NIHSS and ICH score, and larger hematoma volume, are independent risk factors for predicting short-term (30-day) [44]and long-term (90-day) [3]outcomes. In our study, after excluding patients with previous infections, we confirmed that infectious condition within the first 14 days after ICH onset was an independent risk factor for predicting 3-month outcomes. Nam et al. have reported that there is a higher NLR rate of predicted stroke-associated pneumonia (SAP) in patients with acute ischemic stroke who had SAP with poorer clinical outcomes [40]. Our study did not distinguish the infection types of patients with ICH that were related to poorer functional outcomes. Nevertheless, our results provide clues for further large-scale studies exploring the relationship between NLR and infections of patients with ICH, and suggest that early anti-infective therapy may benefit recovery.

In this study, hospitalized patients with sICH who exhibited higher NLR within the first week of admission typically developed a severe disability or died within 3 months. This result is congruent with past findings that a higher NLR during the first week of hospitalization is associated with poorer outcomes in patients with ICH[23]. However, we found that NLR1, NLR3, and NLR7 were not independently predictive of 3-month outcomes. A recent meta-analysis assessed the prognostic role of NLR in patients with sICH and concluded it is time-dependent, with values increasing over the first few days [45]. Conversely, Sun et al. [24] have reported that NLR at admission is not associated with 3-month outcomes post-ICH, possibly due to a single NLR measurement on admission, different risk factors, heterogeneity, and regional disparity. These inconsistent findings should be clarified with further large-scale studies.

There are several limitations of this study. First, it was a retrospective, observational, and single-hospital study with a limited sample size. This led to differences in fluctuant volume and location of hematoma. Therefore, multiple logistic regression analysis may have produced a confounding bias. Second, the cohort age ranged between 18–60 years old, and the proportion of patients with an unfavorable prognosis at 3 months was 26% of 213 individuals. Future studies should investigate associations between immune state and prognosis in a larger number patients with a wider age range. Third, NIHSS score, hematoma volume, mRS scale, and GCS score were not tested using standard machines; therefore, subjective factors may have affected the results even though an average score from two experienced neurologists was obtained for each item. Fourth, due to the complex nature of ICH, our use of a single indicator may not have adequately reflected the full picture. Common residual confounders, including BMI, a history of smoking, drinking alcohol, and blood pressure variability, were not included in the logistic regression analysis. Pro-inflammatory factors, such as lymphocytes and cytokines may also account for residual confounders. Fifth, due to the various time points measured in the present study, the results of the dynamic analysis may not fully represent the whole ICH cohort. Further, immunobiological and functional studies on leukocyte subunits in patients with ICH were lacking in this retrospective observation. In the future, detailed and multidimensional analyses of NLRs as predictive factors for sICH severity and prognosis are required in large-scale, different-term, and multicentric studies.

In conclusion, we observed that dynamic early changes in easily monitored NLRs and ALCs post-hospitalization were positively associated with ICH severity and 90-day outcomes, suggesting that NLR may be used to predict the severity and progression of ICH. Our findings provide insight into the mechanisms underlying post-ICH immunologic responses and may help to optimize clinical management of this condition.

## Supporting information

S1 Table NLRs were not associated with hematoma location. (DOCX)

S2 Table Univariate logistic regression analyses of functional independence (FI, mRS 0–2) at 3 months after discharge. (DOCX)

S3 Table WBC and ANC in logistic regression analysis models for 3-month outcomes. (DOCX)
