# Supplementary material for: Early increased neutrophil-to-lymphocyte ratio is associated with poor 3-month outcomes in spontaneous intracerebral hemorrhage
Source: PLoS One. 2019 Feb 7;14(2):e0211833. doi: 10.1371/journal.pone.0211833 (PMC6366889; doi:10.1371/journal.pone.0211833)
Supplement: S1 Table — (DOCX) [file pone.0211833.s001.docx]

**S1 Table.** NLRs were not associated with hematoma location

|  | Hematoma location (n) | | | Correlation (Kendall’s tau-b, p) |
| --- | --- | --- | --- | --- |
|  | Lobar | Deep | Infratentorial |  |
| NLR0 | 21 | 166 | 26 | 0.093, 0.089 |
| NLR1 | 9 | 54 | 13 | −0.063, 0.495 |
| NLR3 | 10 | 102 | 14 | 0.109, 0.130 |
| NLR7 | 9 | 99 | 15 | 0.103, 0.156 |
| NLR14 | 6 | 87 | 12 | 0.009, 0.912 |

NLR, neutrophil-to-lymphocyte ratio.
