# Supplementary material for: Early increased neutrophil-to-lymphocyte ratio is associated with poor 3-month outcomes in spontaneous intracerebral hemorrhage
Source: PLoS One. 2019 Feb 7;14(2):e0211833. doi: 10.1371/journal.pone.0211833 (PMC6366889; doi:10.1371/journal.pone.0211833)
Supplement: S2 Table — (DOCX) [file pone.0211833.s002.docx]

| **S2 Table.** Univariate logistic regression analyses of functional independence (FI, mRS 0–2) at 3 months after discharge   \| Variables \| OR (95% CI) \| *p* \| \| --- \| --- \| --- \| \| Age \| 0.98 (0.94, 1.02) \| 0.299 \| \| Male sex \| 1.94 (1.00, 3.76) \| 0.051^a^ \| \| Premorbid mRS \| 0.00 (0.00, 0.00) \| 0.999 \| \| Time from onset to sampling, h \| 1.01 (0.94, 1.09) \| 0.767 \| \| Coronary heart disease \| 0.42 (0.12, 1.61) \| 0.205 \| \| Hypertension \| 1.28 (0.65, 2.50) \| 0.477 \| \| Diabetes mellitus \| 1.05 (0.36, 3.03) \| 0.930 \| \| Hyperlipemia \| 0.56 (0.30, 1.06) \| 0.075^a^ \| \| History of stroke \| 0.55 (0.26, 1.16) \| 0.115 \| \| Intensive care unit (ICU) \| 0.05 (0.02, 0.11) \| <0.001^a^ \| \| Endotracheal intubation \| 0.05 (0.22, 0.10) \| <0.001^a^ \| \| Nasogastric feeding tube \| 0.04 (0.01, 0.13) \| 0.001^a^ \| \| Catheter \| 0.00 (0.00, 0.00) \| 0.997 \| \| Operation \| 0.19 (0.10, 0.37) \| <0.001^a^ \| \| Infection \| 0.06 (0.02, 0.17) \| <0.001^a^ \| \| Duration of hospitalization, d \| \| \| \| ≤7 \| \| <0.001^a^ \| \| 7–14 \| 0.18 (0.08, 0.39) \| <0.001^a^ \| \| >14 \| 0.69 (0.31, 1.55) \| 0.364 \| \| Infratentorial \| 0.66 (0.27, 1.65) \| 0.375 \| \| Intraventricular hemorrhage, IVH \| 0.39 (0.20, 0.78) \| 0.008^a^ \| \| Larger ICH volume (>30 cm³) \| 0.07 (0.04, 0.15) \| <0.001^a^ \| \| NIHSS admission score \| 0.77 (0.72, 0.83) \| <0.001^a^ \| \| Glasgow coma scale \| 1.40 (1.28, 1.54) \| <0.001^a^ \| \| White cell counts, 1000/mm³ \| 0.86 (0.80, 0.92) \| <0.001^a^ \| \| Neutrophil counts, 1000/mm³ \| 0.85 (0.80, 0.92) \| <0.001^a^ \| \| Lymphocyte counts, 1000/mm³ \| 1.28 (0.85, 1.93) \| 0.246 \| \| Lymphopenia on admission \| 0.75(0.40, 1.40) \| 0.364 \| \| NLR0 \| 0.94 (0.91, 0.97) \| <0.001^a^ \| \| Monocyte counts, 1000/mm³ \| 0.55 (0.19, 1.63) \| 0.280 \| |
| --- | --- | --- | --- | --- | --- | --- | --- | --- | --- | --- | --- | --- | --- | --- | --- | --- | --- | --- | --- | --- | --- | --- | --- | --- | --- | --- | --- | --- | --- | --- | --- | --- | --- | --- | --- | --- | --- | --- | --- | --- | --- | --- | --- | --- | --- | --- | --- | --- | --- | --- | --- | --- | --- | --- | --- | --- | --- | --- | --- | --- | --- | --- | --- | --- | --- | --- | --- | --- | --- | --- | --- | --- | --- | --- | --- | --- | --- | --- | --- | --- | --- | --- | --- | --- | --- | --- | --- | --- | --- | --- | --- | --- | --- |

ICH, intracerebral hemorrhage; mRS, modified Rankin scale; NLR, neutrophil-to-lymphocyte ratio

^a^*p* < 0.1 were the candidate confounders for multivariate logistic regression analysis
