# Supplementary material for: Early increased neutrophil-to-lymphocyte ratio is associated with poor 3-month outcomes in spontaneous intracerebral hemorrhage
Source: PLoS One. 2019 Feb 7;14(2):e0211833. doi: 10.1371/journal.pone.0211833 (PMC6366889; doi:10.1371/journal.pone.0211833)
Supplement: S3 Table — (DOCX) [file pone.0211833.s003.docx]

**S3 Table**. WBC and ANC in logistic regression analysis models for 3-month outcomes

|  |  | Unadjusted | Adjusted |
| --- | --- | --- | --- |
| WBC, 1000/mm³ | OR (95% CI) | 0.86 (0.80,0.92) | 1.10 (0.96,1.25) |
|  | *p* | <0.001^a^ | 0.182 |
| ANC, 1000/mm³ | OR (95% CI) | 0.85 (0.80,0.92) | 1.13 (0.98,1.30) |
|  | *p* | <0.001^a^ | 0.085 |

WBC, white blood cell; ANC, absolute neutrophil count; OR, odds ratio; CI, confidence interval.

^a^*p* < 0.05, variables associated with 3-month outcome in single factor analysis.
